# Mechanisms of Silicon-Mediated Amelioration of Salt Stress in Plants

**DOI:** 10.3390/plants8090307

**Published:** 2019-08-27

**Authors:** Boling Liu, Prabhakaran Soundararajan, Abinaya Manivannan

**Affiliations:** 1 School of Life Sciences, Qufu Normal University, Qufu 273165, China; 2 Department of Agricultural Biotechnology, National Institute of Agricultural Sciences, RDA, Jeonju 54874, Korea; 3 Vegetable Research Division, National Institute of Horticultural and Herbal Science, Rural Development Administration, Jeonju 55365, Korea

**Keywords:** agriculture, abiotic stress, nutrients, physiology, photosynthesis, Silicate

## Abstract

Silicon (Si), the second most predominant element in the earth crust consists of numerous benefits to plant. Beneficial effect of Si has been apparently visible under both abiotic and biotic stress conditions in plants. Supplementation of Si improved physiology and yield on several important agricultural and horticultural crops. Salinity is one of the major abiotic stresses that affect growth and yield. The presence of high concentration of salt in growing medium causes oxidative, osmotic, and ionic stresses to plants. In extreme conditions salinity affects soil, ground water, and limits agricultural production. Si ameliorates salt stress in several plants. The Si mediated stress mitigation involves various regulatory mechanisms such as photosynthesis, detoxification of harmful reactive oxygen species using antioxidant and non-antioxidants, and proper nutrient management. In the present review, Si mediated alleviation of salinity stress in plants through the regulation of photosynthesis, root developmental changes, redox homeostasis equilibrium, and regulation of nutrients have been dealt in detail.

## 1. Introduction

Salinity is one of the predominant abiotic stresses that affect agricultural production. The adverse effects of salinity have damaged at least 20% of crop cultivation around the world [1]. Upon the exposure to high saline conditions, the water absorption rate of plants is reduced drastically. This affects inter- and intra-cellular water level and inhibits the cell expansion followed by decline in stomatal activity. The long term exposure of salinity results in substantial ionic and oxidative stresses that are caused due to the higher amount of NaCl influx [2]. Ionic and osmotic imbalances under salt stress condition impairs the growth and development of plants [3]. Accumulation of salts decreases the concentration of photosynthetic pigments such as chlorophyll and carotenoid followed by the inhibition of ribulose-1,5-bisphophate and degrades the photosynthetic apparatus. Consequently, generation of higher amounts of reactive oxygen species (ROS)surpasses the level of scavenging rate [4]. Moreover, excessive ROS hinders the transpiration, nutrient uptake, damages vital macromolecules such as nucleic acids, proteins and lipids, collapses membrane integrity, and other vital metabolisms [5]. Interference of NaCl in the protein synthesis, activities of enzymes, and photosynthesis leads to the chlorosis, necrosis, and premature senescence of older leaves [3]. To overcome the salt stress plants need to improve ion exclusion, osmotic tolerance, redox homeostasis, and efficient photosynthesis. In order to combat the adverse effect of salinity stress in plants various researches have been initiated and ongoing in myriads fields of plant biology.

Silicon (Si) is the second most abundant element in the earth crust. Previously, Si was considered as beneficial but non-essential element. Recently, the International Plant Nutrition Institute included Si as “quasi-essential” element. Plants uptake Si in the form of soluble silicic acid [Si(OH)_4_] under less than 9 pH [6]. Supplementation of Si has been proved as beneficial to plants in several ways such as increasing yield, resistance against diseases, and alleviation of abiotic stresses. The amendment of Si nutrition has been reported against various stresses including, salinity [7], higher temperature [8], powdery mildew [9], hyperhydricity [10], and herbivores [11]. In general, Si mediated stress resistance mechanism can be attributed to the following physiological improvements in plants; enhancement of growth and biomass, management of essential nutrients, maintenance of structural rigidity, increased photosynthesis efficiency, lodging resistance, balancing the ion homeostasis, activation of antioxidant system in plants, elicitation of secondary metabolites related to stress resistance, and regulation of genes involved in various physiological processes [12]. Thus, the numerous merits of Si have been exploited in diverse crops to increase the yield and stress tolerance. Although, several reports have evidenced the merits of Si nutrition, the exact molecular mechanism behind the stress alleviation is still under study. However, in the current review the available mechanisms of silicon to alleviate salinity stress have been summarized.

### Stress Tolerance Imparted by Silicon

Under salinity condition, Si decreases the apoplastic transportation of Na^+^ and Cl^−^ [13,14]. In Si accumulators, formation of double-cuticle layers by amorphous-Si increases the photosynthetic rate and decreases the evapo-transpiration. Further, Si improves the rigidity and erectness of leaves to enhance the photosynthetic canopy [15]. Photosynthesis pigment degradation was efficiently prevented by the application of Si. Photosynthesis-related proteins such as photosystem-I, -II, RubisCO, and other chloroplast-related proteins were regulated by the Si during the salinity and hyperhydric condition [10,16]. Several studies reported that Si modulated the redox-homeostasis mechanism. Supplemented Si regulated the activities of antioxidant enzymes such as superoxide dismutase (SOD), catalase (CAT), guaiacolperoxidase (GPX), and ascorbate peroxidase (APX) to overcome the redox imbalance [17,18,19]. In addition, lower molecular non-enzymatic antioxidants such as glutathione and proline content under the stress condition was improved by the Si supplementation [20].

Recent study showed that biochars made from bamboo combined with the Si (Si-biochar) effectively reduced the arsenic (As) bioaccumulation in spinach by 37.7%. Additionally, Si improved the dry biomass of spinach to 67.7% [21]. Under As stress condition, expression of Si transporters such as *Lsi1* and *Lsi2* was increased in *Oryza sativa* along with glutaredoxin (GRX) and glutathione-S-transferase (GST) [22]. A study conducted between the drought resistant and drought sensitive lines of tomato showed that Si application resulted on different response in both genotypes. In tolerant line, Si improved sulfur (S) and ammonium (NH_4_^+^). This leads to the higher synthesis of amino acids such as arginine, methionine, serine, and glycine. Moreover, in drought sensitive tomato the augmentation of Si improved the proline and gamma-aminobutyric acid (GABA) levels. Enhancement of GABA reduced the level of glutathione oxidized glutathione (GSSG) to oxidized glutathione (GSH) ratio and maintains equilibrium in redox homeostasis [23]. Over expression of *Lsi1* into the rice under cold stress efficiently improved several physiological processes [24]. Freezing tolerance of Si studied in *Vitis* *vinefera* showed that foliar application was more effective than the soil. Freezing stresswas overcome by Si in improving PSII, carotenoid, and membrane integrity [25]. Freezing resistance could be attributed to the higher antioxidant capacity in the Si treatment of *Pistaciavera* [26]. Application of Si reduced the cadmium translocation from root to shoot in *Nicotiana* *tobacum*. Reduction of Cd uptake could contribute for lowering the potential health risks of Cd contamination [27]. The activities of enzymatic and non-enzymatic antioxidants activities were highly modulated in wheat under various abiotic stress conditions such as drought, salt, and Cd stress [28]. Wu et al. [29] reported that Si decreased the Cd uptake by modulating the root endodermal suberin development in wheat. It is correlated with higher expression of Cd efflux-related gene *Triticum aestivum transmembrane 20 (TaTM20*). Si efficiently regulated the sulfur deficiency and osmotic stress on barley by modulating the *sulfur transporter*(*HsST1*) and abscisic acid (ABA) metabolism related genes such as *glyceraldehydes-3-phosphate dehydrogenase* (*GAPDH*), *cyclophilin* (*CYC*), and *ADP-Ribosylation factor* (*ADP-RF1*) [30]. Similarly, Si treated *Poaannua* seedlings displayed more tolerance against Cd toxicity [31]. Influence of Si under osmotic stress on starch metabolism, glycolytic, and TCA pathways has been reported in *Hordeum vulgare* [32]. Likewise, supplementation of Si in the in vitro culture medium ameliorated hyperhydricity. Recently, Manivannan et al. [33] reported that inclusion of potassium silicate (K_2_SiO_3_) and calcium silicate (CaSiO_3_) on different concentrations along with 1.0 mgL^−1^ of 6-benzyladenine and 0.5 mgL^−1^ of indole-3-acetic acid induced shoot proliferation of carnation. Modulation in the expression of SOD, GPX, CAT and protection of stomatal damage under Si treatment could be important key factors to recover the hyperhydricity. In addition to that up regulated proteins in Si treatment was categorized into ribosomal binding, oxido-reduction, hormone/cell signaling, metal/ion binding, defense, and photosynthesis [10]. In *Rosa* *hybrida*, Si nutrition and salinity stress altered the expression of proteins associated with various physiological and developmental process and vital metabolism [20]. In detail, the supplementation of Si regulated the levels of proteins involved in the redox homeostasis, transcription and translation, lipid metabolism, signaling, carbohydrate metabolism, metal ion binding and transportation, cell wall synthesis, and terpene metabolism. Overall, Si mediated stress amelioration is a complex mechanism involving a cascade of physiological and metabolic reactions which need a deeper insight to unravel the exact molecular strategy implemented by Si to impart stress resistance in plants.

**Figure 1:**
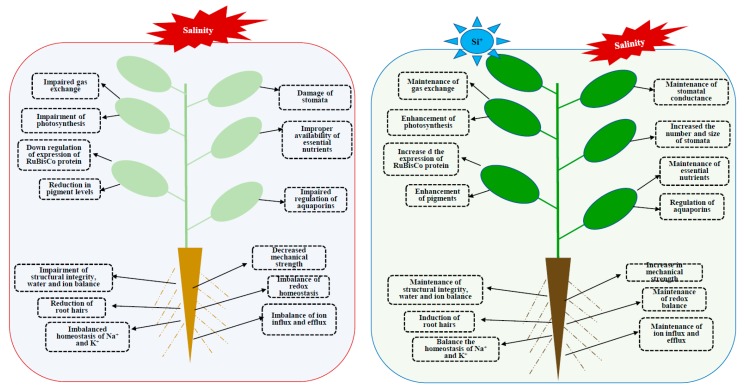
Schematic representation of damages caused by salinity stress and the Si mediated mitigation of salinity in plants.

Taken together, salinity causes deleterious effect to the plant growth and development whereas Si supplementation showed improved tolerance against salt stress. Therefore, the present endeavor reports key mechanisms on the role of Si in overcoming salt stress such as restriction of Na^+^/Cl^−^ uptake via root, improvement of photosynthetic process, maintenance of redox homeostasis, and effective management of essential elements. The overall schematic illustration of Si mediated enhancement son plant under salinity conditions have been illustrated in Figure 1.

## 2. Mechanism to Restrict Na^+^/Cl^−^ Uptake via Root

### 2.1. Structural Modifications Imparted by Si to Combat Salt Stress

In roots, Si deposition on the inner tangential and radial cell walls enhances the mechanical strength of the plant [34]. However, the silicification of cells in most cases resulted in the restriction of cell elongation. The biomass enhancement by Si supplementation in rice imparted mechanical hardness to the tissues and aided in the maintenance of optimal water balance [35]. Recent report by Soundararajan et al. ([20]) has illustrated the structural integrity maintenance of root cell wall by Si in salt stressed *R. hybrida*. According to the report, Si supplementation resulted in the induction of root hairs could have supported for an effective mineral nutrient absorbance from the hydroponics medium [20]. Moreover, the higher occurrence of root hairs aids in the uptake of water and helps to combat the salinity, drought, and heavy metal stress [36]. Further in-depth studies are necessary to identify the molecular mechanism behind the Si mediated induction of root hairs and morphological modifications in root structure.

### 2.2. Aquaporin in Mineral Uptake under Silicon Nutrition and Stressed Condition

Under salinity stress, the primary tolerance have been achieved by maintaining the lower intracellular Na^+^ concentration either by increasing the Na^+^ efflux or decreasing the influx of Na^+^ ion [37]. Generally, Na^+^ enters the plant system passively through roots by non-selective cation channels and Na^+^ transporters like high affinity K^+^ transporters (HKTs) [38]. Previous studies have identified the Si-mediated selective ion uptake and management of Na^+^/K^+^ ion channels. In sugarcane, the addition of Si hindered the uptake of Na^+^ but increased the accumulation of K^+^ [39]. Similar activities of Si have been reported in roses [20], aloe [40], and zinnia [19]. Apart from the above mentioned mechanism, Si have also been reported to regulate the levels of both macro and micro nutrients under salt stress [36].

Plant transporters in root play a vital role in the uptake of minerals from the soil or hydroponics medium. The uptake of minerals and water are highly inter-related since both involve transporters. Aquaporin are important transporters that regulate the uptake and transportation of water and minerals across the cell membranes [41]. Plant aquaporin belongs to membrane intrinsic proteins (MIP) family of transmembrane proteins. Based on the phylogenetic distribution, subcellular localization, substrate selection, length of the sequence, and function the aquaporin are classified into subfamilies such as plasma membrane intrinsic proteins (PIP), tonoplast intrinsic proteins (TIP), nodulin26-like intrinsic proteins (NIP), small basic intrinsic proteins (SIP), uncharacterized intrinsic proteins (XIP), and hybrid intrinsic proteins (HIP) [42]. Apart from water uptake, aquaporins are involved in the facilitation of mineral transportation. Previous studies have demonstrated the participation of aquaporin in the regulation of solute transports such as urea, ammonia, hydrogen peroxide, lactic acid, and silica acid [43]. The substrate selectivity of an aquaporin is primarily depend on the following factors, occurrence of NPA motifs for the exclusion of H^+^ and a filter consisting of an aromatic/arginine region in the pore area [44].

Various isoforms of NIP subfamily such as NIP1 is highly permeable to water, whereas NIP2 aids in the transportation of metalloids and Si, andNIP3 function as boric acid transporter [43]. Si influx and efflux transporters such as *Lsi1* and *Lsi2* belonging to NIP subfamily have been identified by Ma et al. [44] in rice. These transporters are one of the major factors that determine the transportation, distribution, and accumulation of Si. During salinity stress, in the presence of Si the cellular water balance has been regulated by PIP subfamily. On the other hand, NIP subfamily involved in the uptake of Si [45]. Si regulated the PIP aquaporin expression and restored the hydraulic conductance in roots of Sorghum under short term salt stress [46,47]. Moreover, in plants the deficiency of nutrients under stressed condition correlated with the decrease in water uptake. This process illustrates the vital role of aquaporin in nutrient management. For instance, the significant role of aquaporin in the homeostasis of nutrients include the provision of support for the passive movement of nutrients along with water and channeling the apoplastic/symplastic water flow within tissues [48]. In addition, Martines-Ballesta et al. [49] reported the nutrient balance by the synergistic regulation of aquaporins along with ATPase and Ca-ATPase. Numerous factors influence the activity of aquaporin such as levels of abscisic acid and calcium, free radicals and hormones like ethylene [50,51]. Among these factors, even a small variation in the free radical contents affect the aquaporins [52]. Higher H_2_O_2_ levels generated by salinity stress prevented the activity of aquaporin by obstructing the oxidant gating, post-translational modification like phosphorylation, and aquaporin re-localization [52]. However, the molecular rationale behind the aquaporin interactions and mineral nutrients under stressful environment are still under study. Several researches have been progressed towards deciphering the impacts of Si nutrition to plants upon stressed conditions however only a few studies have dealt with the molecular aspects of Si mediated gene regulations in plants [53,54]. In Sorghum, the inclusion of Si resulted in the improvement of water uptake by enhancing the function of aquaporin by the up-regulation of aquaporin genes such as *SbPIP1;6*, *SbPIP2;2*, and *SbPIP2;6* in root [47]. According to Gao et al. [55], Si increase the aquaporin related gene expression which in turn attenuate the lethal effects of Na^+^ ion upon high salinity condition.

It has been evident that the presence of Si increased root growth and balances the homeostasis of Na^+^ and K^+^ ratio. The substantial enhancement of genes associated with aquaporin by Si might lead to the improvement of water status in plants under stressful environment. Therefore, amendment of Si develops the restoration of water status and mineral ion balance which could promote the reclamation of plants from ionic and osmotic stresses.

## 3. Leaf Physiology and Photosynthesis

Photosynthesis is the primary metabolism of plant that provides energy for the growth and development. It can be easily affected by any kind of stress including salinity. Altered photosynthesis disrupts the carbon availability. Improper photosynthetic process leads to accumulation of ROS. Degradation of chlorophyll affects the overall photosynthetic process. Oxidative stress causes the damage in cellular organelles. In severe cases, leaf scorch and curling of leaves results in plant death. Prevention of photosynthetic damage is one of the vital steps to overcome the salinity stress [56,57].

### 3.1. Improved Photosynthetic Efficiency

Decline in plat metabolism and biomass under salinity stress was correlated with the decreased photosynthesis rate [58]. However, previous reports showed that exogenous treatment of Si improved the photosynthetic gas exchange process on several plants including tomato [59,60], sorghum [61], maize [62], tobacco [63], and pumpkin [64]. Exogenous application of Si in low levels (0.8 and 1.6mM) significantly enhanced the photosynthetic rate and water-use efficiency in Maize [62]. According to Zhu and Gong [65] Si restricts the Na^+^ accumulation and improved K^+^ uptake as well as maintenance of osmotic balance helps to enhance photosynthetic process under salt and drought condition. Ability of Si to increase the photosynthetic pigment, chlorophyll concentrations, and protect the photochemical apparatus in saline condition was reported in *Spartina* *densiflora* [66], *Abelmoschus* *esculentus* [67], and *Capsicum annuum* [7]. Furthermore, Manivannan et al. [7] described that the abundance of carbon fixation and photosynthetic-related proteins were higher in Si supplemented NaCl treatment. In addition, Muneer et al. [16] investigated the chloroplast proteins of tomato under salinity stress conditions with or without Si. The results showed that total chlorophyll and carotenoid content, net-photosynthesis rate, stomatal conductance, and transpiration were increased in the salt treatments with Si supplementation. Furthermore, addition of Si alleviated the reduction in cytochrome *b6/f* and the ATP-synthase complex under salt stress. Recently, Soundararajan et al. [68] also showed that Si increased the abundance of photosynthesis and energy metabolism-related proteins in *R*. *hybrida* under salt stress. Beneficial effects and higher yield in Si treatment can be correlated with the ability of Si to activate genes/proteins involved in metabolic process [69].

### 3.2. Stomata and Chloroplast Movement

During stress conditions improper gas exchange and reduction of CO_2_ accumulation was mostly due to damage/malfunctioning of stomata [70]. However, several researches have showed that Si treatments maintain a higher stomatal conductance and transpiration rate in plants [47,56,62,67]. Parveen and Ashraf [62] reported that application of Si in the rooting medium improved stomatal conductance and leaf sub-stomatal CO_2_ assimilation rate under saline condition in *Sorghum bicolor* [56]. Exogenous application of Si improved the stomatal conductance due to the increase in the stomata number and stomata size in salinity stressed okra (*Abelmoschus* *esculentus*) plants [67]. Application of Si maintain a higher stomatal conductance due to the improvement of the leaf water content [47]. In *Capsicum annuum* most of the stomata were closed in NaCl treatment however, stomata remained normal in NaCl with Si [7]. It has been demonstrated that the Si enhanced the expression of the zinc finger protein-160, which controls the movement of stomatal aperture to avoid water loss under saline condition [71].

### 3.3. Photosystem I and II

Furthermore, the reduction of photosynthesis of plants under salt stress could be due to the damage of the photosynthetic apparatus, resulting in the decrease in PSII efficiency [72]. It has been reported that salinity stress inhibits PSII [73,74]. However, reduction of PS I and II could be alleviated by the application of Si. Muneer et al. [16] showed that PSI and PSII complexes were nearly absent with high salt stress, but Si addition helped the plants to retain the protein complexes in tomato plants. In addition, the expression of PSI-monomer/cytochrome *b6f* was decreased under salinity stress, while the reduction of PSI-monomer/cytb6f was alleviated when Si supplemented with NaCl to the tomato seedlings. Matios-Naranjo et al. [75] showed that addition of Si under salinity condition improved pigment concentration and PSII efficiency in the halophytic grass *Spartina* *densiflora*. Khan et al. [76] reported that Si is a beneficial nutrient to increase the efficiency of photosystem II in maize under salinity stress. Soundararajan et al. [68] showed that decrease in the expression of key proteins in photosynthesis and energy metabolism such as NAD(P)H-quinone oxidoreductase enzyme, which regulates the turnover of the PSII reaction center, were improved upon the addition of Si under NaCl treatment in rose.

Firstly, Si supplementation prevents the degradation of photosynthetic pigments such as chlorophyll and carotenoid. Secondly, damage of photosynthetic apparatus including stomata was avoided in the Si treated NaCl plants. Finally, enhanced expression of PSI, PSII, and other photosynthesis-related proteins were associated with the photosynthesis and physiological improvement.

## 4. Redox Homeostasis

Usually ROS are generated during the metabolic processes from the organelles such as mitochondria, chloroplast, peroxisomes, glyoxysomes, and plasma membrane. In the controlled level ROS act as signaling molecules [77]. Active involvement of antioxidant enzymes and low molecular antioxidants detoxify the ROS to keep it under controlled level. Under stress conditions, plants experience oxidative stress due to the higher lipid peroxidation rate, thiobarbituric acid reactive substances (TBARS), and enormous generation of ROS. Especially, uncontrolled superoxide anion (O_2_^−^) and hydrogen peroxide (H_2_O_2_) are able to damage lipids, proteins, and nucleic acids.

### Si Mediated Maintenance of Redox Homeostasis under Salinity Stress

Previous studies suggest that to cope up with the salt stress, plants need to maintain the equilibrium between the rates of ROS generation and its scavenging. At low concentration, ROS can act as signaling molecules whereas in higher level ROS damages the cellular components. Efficient antioxidant system was rendered by the Si in the several plants by regulating the SOD, GPX, APX, and CAT. Primarily SOD catalysis the dismutation of superoxide into hydrogen peroxide and oxygen. Later enzymes such as CAT, GPX, and APX detoxify the H_2_O_2_ into water [78]. Even though, O_2_^−^ has the very limited half-life (2μs), reduction of O_2_^−^ into H_2_O_2_ can travel long distance as it has half-life of about 1 ms. Other than above two, ^•^OH molecules also formed during the excessive generation of O_2_^−^ and H_2_O_2_. Formation of ^•^OH radical is mediated by iron in the Haber-Weiss and Fenton reactions. Therefore, uncoupled electron of ROS could be able to react with other metabolites and cross-link with the essential metabolic reactions. These excessive generations affect the normal growth and development of plants. Numerous studies have evidenced the Si mediated maintenance of redox homeostasis [7,8,16,18].

Effect of Si on the maintenance of ROS varied between plant species and treatment. In *Salvia* under temperature stress CAT level was decreased [8] whereas in wheat the activity of CAT was increased under salt stress [79]. In *Brassica napus*, Si mitigated the oxidative damage caused by higher H_2_O_2_ and TBARS. Methylglyoxal (MG) toxicity aroused due to higher TBARS was inhibited with the induced expression of glyoxalase I (Gly I) and Gly II. Oxidative stress mitigation was associated with the higher activities of antioxidant enzymes such as APX, MDHAR, DHAR, GR, GST, GPX, and CAT [80]. In another study, Si protected the photosynthetic pigments and reduced the oxidative stress by activating antioxidant enzymes, increased ascorbate-glutathione (AsA-GSH) pool, proline, and glyoxalase systems in drought stressed *B. napus* [81]. Similar result was observed in *B. juncea* under salt stress with Si treatment alone or in combination with 24-Epibrassinolide (EBL) [82]. Amelioration of salt stress related damages such as leaf scorching and stomatal malfunction in the *R. hybrida* was correlated with the improved photosynthetic efficiency, reduction in the degradation of photosynthetic pigment, lesser lipid peroxidation rate, accumulation of ROS, and enhanced antioxidant metabolism [20]. Biofortification and prevention of water loss in the salt tolerant and salt sensitive cultivars of rice was observed in Na_2_SiO_3_ treatment. Higher activities of ascorbate-glutathione cycle related enzymes such as glutathione reductase (GR), APX, GPX, and GST boosted antioxidant defense mechanism. Effect of Si on salt-sensitivity cultivar was distinct than the salt-tolerant cultivars [83].

Although regulation of antioxidant enzymes and low molecular weight antioxidant are varied between species as well as treatments, Si-mediated redox homeostasis is critical toretain ROS level and essential metabolic process of plants.

## 5. Essential Elements Management by Si under Salt Stress

The Si mediated alleviation of stress involves the effective managements of essential elements in plant system. Upon salinity stress the proper nutrient channeling is affected and leads to the imbalance in the macro and micro elements. Several studies have reported the positive role of Si in the nutrient management.

Potassium, the monovalent cation with high mobility involved in the maintenance of osmotic strength is essential for the plant growth and yield [84]. In addition, K plays a key role in the synthesis of proteins, photosynthesis, and activities of glycolytic enzymes in plants. Further, the potassium metabolism is essential for the maintenance of carbohydrate synthesis and nitrogen assimilation in plants. Higher levels of Na under salinity stress hinder the acquisition of K and result in the deficiency. The augmentation of Si modulated the competitive uptake between Na and K and improved the intercellular distribution of K in salt stressed wheat [85]. In blueberry, the Si deposition in the lower epidermis of the stomata and enhancement of K has been considered as the vital mode of stress alleviation [86]. Moreover, ABA formed during the salt stress seals the stomata during the increased K^+^ efflux from the guard cells [87]. The Si-mediated movement of stomata could be associated signal perception, electrochemical gradient, osmotic adjustment, and ion balance [88]. The size and structural similarity between K^+^ and Na^+^ leads to the competitive uptake since the K^+^ transporters lacks discrimination between the K^+^ and Na^+^ ions. However, the Si application increased the selectivity between K^+^ and Na^+^ ions [89]. Moreover, Si treatment increased the K accumulation in K-deficient condition than the K^−^ sufficient environment [90].

In similar manner with potassium, the element calcium (Ca) is important for the proper maintenance of structural membrane integrity and plays a vital role in the senescence process [91]. Irrespective of the low mobility of Ca, even a slight modulation in the active pools of Ca within the cytoplasm reflects in the major physiological processes. In addition, Ca plays a major role in the cell division and cell elongation processes. In plants, Ca is uptake by apoplast bound to the outer surface of the plasma membrane in exchangeable form. Higher concentration of Na inhibits the absorption of Ca which has been reversed by the addition of Si in rice [92]. Moreover, the synergistic improvement of both K and Ca by Si in the salinity stress condition could facilitate the balance of permeability and selectivity of cell membrane under stressed conditions [93]. In salt stressed barley, Ca deficiency resulted in the higher leakage of solutes from the cells which has been ameliorated by the supplementation of Si [94]. Moreover, the cytosolic Ca is involved in the process of induction of stress-tolerance genes under salinity condition. Thus, the Si mediated enrichment of Ca could assist in cell developmental process particularly under stressed environment.

Nitrogen (N) is an inevitable element and acts as the predominant factor for the plant growth [95]. Under extreme salt condition, the uptake of N is hindered [96]. Deficiency of N reduces photosynthesis and cellular expansion [97]. In rice the salt mediated deficiency of N has been alleviated by the application Si which increased the N level and chlorophyll content [98]. Likewise, phosphorous (P) is vital for the energy metabolism, structural component of nucleic acids, and lipids. High concentration of salt decreased the level of P availability due to the high ionic strength exerted by Na^+^ and Cl^−^ ions [99]. Whereas the application of Si balanced the P levels under stressed condition, for instance Si channeled the utilization of internal P during the deficient conditions and on the other hand Si inhibits the excess accumulation of P [100]. Apart from the abovementioned macronutrients, the Si also facilitated the management of micronutrients such as boron, iron, zinc, copper, and manganese in diverse crops under salinity stress condition.

As macro and micro nutrients are necessary for the plant metabolism, lesser availability of essential elements inhibits the growth and development. However, restriction of Na^+^ and subsequently competitive uptake of other ions in Si treatment could support the maintenance of essential elements under salinity condition.

## 6. Conclusions

Application of Si consists of numerous benefits to plants particularly in the alleviation of salinity stress by improving photosynthesis, redox balance, and nutrient management. Stimulation of root growth and maintenance of cell wall integrity withstands the selective permeability in plants. Meanwhile, prevention of damage of photosynthetic apparatus and higher photosynthetic rate sustain the availability of carbohydrate. Proper redox-homeostasis equilibrium avoids the cross-reaction of excessively generated ROS with other key metabolism. Restriction in Na uptake, counter intake of K, Ca, P, and maintenance of water influx on Si treatment under salt stress are associated with the efficient regulation of essential elements for overall physiological improvement.

Due to its wide merits, the addition of Si as a supplemental nutrient in the contemporary agricultural practices such as soil-less cultivation system has been recognized in several areas. However, in-depth molecular rationales behind the Si mediated stress mitigation have to be addressed in future. Recent advancements in the omics technologies could aid in the understanding of Si biology in various plants particularly under stressed conditions. Further, researches determining the possible mechanisms and ways for innovative incorporation of Si in the culture/growth media for the improvement of plant yield as well as stress tolerance could benefit the cultivation of agricultural and horticultural crops
